# Monthly Summary

**Published:** 1892-08

**Authors:** 


### Monthly Summary.

NEURALGIA.—For six months a lady, at frequent intervals, had terrible neuralgia of the shoulder, going down to the forearm. I asked her if her teeth had been examined, and she said that they had never been suspected by her physician as the cause of the trouble. I found under the free margin of the gum, on the buccal side of the wisdom-tooth, a cavity that penetrated to the pulp chamber. It was such a difficult place to get at, and she had suffered so long, I advised her to have it out, and after the extraction of the tooth the neuralgia disappeared from the shoulder.

I have seen other cases of neuralgia where, apparently, a very slight thing caused it all. I remember one case of a patient suffering with intense neuralgia, where there was no cavities in the teeth, except what had been well filled, but there was a noticeable recession of the gums, which I decided was the cause, from their hyper-sensitiveness. The exposed parts of the teeth were treated with a little nitrate of silver, which removed the sensitiveness and also the neuralgia.—*Dr. Smith, in International.*

